# Ectopic Fat Deposition on Insulin Sensitivity: Correlation of Hepatocellular Lipid Content and* M* Value

**DOI:** 10.1155/2016/3684831

**Published:** 2016-11-01

**Authors:** Beverly S. Hong, Ying Li, Shuiqing Lai, Juan Liu, Hongyu Guan, Weijian Ke, Xiaoying He, Yanbing Li

**Affiliations:** ^1^Department of Endocrinology and Diabetes Center, The First Affiliated Hospital of Sun Yat-sen University, Guangzhou, China; ^2^Department of Hematology, The First Affiliated Hospital of Sun Yat-sen University, Guangzhou, China; ^3^Department of Endocrinology, Guangdong General Hospital, Guangdong Academy of Medical Sciences, Guangzhou, China

## Abstract

*Purpose*. This study aimed to explore the relationship among insulin sensitivity and ectopic fat depots in participants with different glucose status.* Methods*. Fifty-nine men and women were enrolled in this study: 29 with normal glucose tolerance (NGT), 17 with impaired glucose tolerance (IGT), and 13 with type 2 diabetes mellitus (T2DM). All participants underwent a hyperinsulinemic-euglycemic clamp to assess the insulin sensitivity index (*M* value) and magnetic resonance imaging to measure the hepatocellular lipid content (HCL), skeletal muscle fat content including intramyocellular lipid (IMCL) and extramyocellular lipid (EMCL) of tibialis anterior (ta), and soleus muscle (sol).* Results*. The *M* value of NGT group was higher than those of IGT and T2DM groups (*P* = 0.001). Participants with T2DM had the highest HCL and IMCL (ta) compared with those in NGT and IGT groups (*P* = 0.001). The *M* value had an inverse relationship with HCL (*r* = −0.789, *P* = 0.001), IMCL (sol) (*r* = −0.427, *P* = 0.002), and IMCL (ta) (*r* = −0.419, *P* = 0.002). Stepwise linear regression analysis showed that HCL (standardized *β* = −0.416; *P* = 0.001) had an independent relationship with *M* value.* Conclusions*. Hepatocellular lipid content deposition happens earlier than skeletal muscle fat deposition. HCL is an independent risk factor for insulin resistance and may be used to evaluate the risk of developing T2DM as a noninvasive marker of insulin sensitivity index.

## 1. Introduction

Insulin resistance leads to impaired glucose absorption, promotes hepatic gluconeogenesis, and is associated with the metabolic syndrome, especially with its effects on the liver and muscle [1]. Insulin resistance promotes hydrolysis of triglycerides and release of free fatty acids from adipose tissues, reduces glucose uptake in muscle cells, impairs glycogen synthesis, and increases gluconeogenesis, lipogenesis, and synthesis of CRP in the liver [2, 3].

Triglycerides in the liver and skeletal muscle promote peripheral and hepatic insulin resistance, thereby contributing to the pathogenesis of type 2 diabetes mellitus (T2DM) [4, 5]. Diabetics have a greater muscle triglyceride (MT) content than individuals with impaired glucose tolerance (IGT) and normal glucose tolerance (NGT), independent of the total body fat percentage [6]. However, some studies showed no association between MT levels and glycemic control in obese individuals [7]. Another study showed that MT content is associated with hepatic, but not peripheral, insulin resistance [4].

Studies on the relationship between insulin sensitivity and lipid content have shown inconsistent results. Some but not all studies have shown correlation between intramyocellular lipid (IMCL) content with measurements of insulin resistance [8–10]. Conversely, some studies have shown a direct relationship between intrahepatic triglycerides and IMCL in the soleus muscle, but not the tibialis anterior [11, 12]. A relatively minor loss of body weight (3%) was accompanied by a major reduction in visceral fat mass (12%) and in LF content (33%) [13].

In this study, the fat depots of liver and skeletal muscle with insulin sensitivity were assessed in study participants, aiming to explore the relationship between these parameters.

## 2. Methods

### 2.1. Study Population

Between January, 2013, and August, 2014, 59 individuals (males: 30) were included in this study: 29 with normal glucose tolerance (NGT), 17 with impaired glucose tolerance (IGT), and 13 with type 2 diabetes mellitus (T2DM). The participants with T2DM had the oral hypoglycemic drugs including metformin or acarbose. All participants underwent a hyperinsulinemic-euglycemic clamp (*M* value) and magnetic resonance imaging (MRI) to calculate hepatocellular lipid content (HCL) and ^1^H-magnetic resonance spectroscopy to measure intramyocellular lipid (IMCL) and extramyocellular lipid (EMCL) of tibialis anterior (ta) and soleus muscle (sol). The study protocol was approved by the ethics committee at The First Affiliated Hospital of Sun Yat-sen University, Guangzhou, China, and all participants provided written informed consent. The study was performed in compliance with the Declaration of Helsinki and Good Clinical Practice.

### 2.2. Measurements

The weight and height of all participants were measured to calculate the BMI (kg/m^2^). The waist circumference (WC) was measured at the midpoint between the lowest rib and the uppermost lateral border of the right iliac crest and hip circumference (HC) was measured at their widest point to calculate the waist-to-hip ratio (WHR). Blood pressure was measured using a mercury sphygmomanometer at a resting state. All blood samples were taken in the morning following an overnight fast of at least eight hours. The following were measured: fasting plasma glucose (FPG), 30-minute plasma glucose (PG30 m), two-hour plasma glucose (PG2 h), hemoglobin A1C (HbA1c), fasting insulin (FINS), serum total cholesterol (CHOL), triglycerides (TG), high-density lipoprotein cholesterol (HDL-c), low-density lipoprotein cholesterol (LDL-c), alanine aminotransferase (ALT), aspartate aminotransferase (AST), and gamma-glutamyl transpeptidase (GGT). The estimated glomerular filtration rate (eGFR) values were calculated from creatinine levels using the CKD-EPI formula.

### 2.3. *M* Value, *β* Cell Function, and Insulin Sensitivity

A hyperinsulinemic-euglycemic clamp was used after an eight-hour fast on a different day. In brief, insulin diluted in 0.9% saline was infused intravenously at a constant rate of 80 mU/m^2^ per minute for 2 hours. Plasma glucose was clamped at 5.6 mmol/L with a variable-rate infusion of 20% dextrose based on the arterialized plasma glucose value, which was measured every five minutes. The insulin levels were maintained at 86.5 (68.5–106.2) *μ*U/mL. Peripheral insulin sensitivity (*M* value) was calculated by dividing the insulin-stimulated glucose disposal rate by the steady-state plasma insulin concentration during the last 30 minutes of the clamp [14].

Homeostasis model assessment of insulin resistance (HOMA-IR) values was calculated as FPG (mmol/L) × FINS (*μ*U/mL)/22.5. Homeostasis model assessment-*β* (HOMA-*β*) values were calculated as 20 × FINS (*μ*U/mL)/[FPG (mmol/L) − 3.5]. Insulin activation indices (IAI) values were calculated as 1/[FPG (mmol/L) × FINS (*μ*U/mL)]. Quantitative insulin sensitivity check index (QUICK) values were calculated as 1/[log FPG (mg/dL) + log FINS (*μ*U/mL)].

### 2.4. Body Fat Composition, HCL, IMCL, and EMCL

The body fat ratio (BFR), fat mass (FM) index, and lean mass (LM) index were measured using human body composition analyzer (Tanita MC-180, Japan). Electrodes were attached to parts of the body and a small electric signal was circulated. The impedance or resistance to the signal was measured as it traveled through the water found in muscle and fat.

Subjects underwent an upper-abdominal coil MRI (3-Tesla whole-body scanner; SIEMENS 3.0 T MAGNETOM Verio) examination that involved an initial set of localizer images and the T1 volumetric interpolated breath hold examination (VIBE) Dixon sequence to calculate HCL [15]. All subjects were imaged in supine position and carefully instructed to hold breath during end inspiration to ensure consistency among subjects. The parameters of the images were TE (echo time), 2.5 ms/3.7 ms and TR (repetition time), 5.47 ms. Four images were generated at the same time within a single breath hold, including in-phase and out-of-phase, as well as fat and water phase images. HCL map was generated by combining the MR images obtained during both fat phase and water phase of each subject. This was done by using a plug-in algorithm created under MATLAB platform (MATLAB r2011b, MathWorks, American), using the following equation: HCL = *F*/(*W* + *F*)*∗*100% (*F*: fat volume; *W*: water volume). Two radiologists manually placed an irregular-shaped region of interest (RIO) covering the entire liver in 21 consecutive slices (max-area centered) of each patient. The mean HCL in the ROI of each subject was recorded.

The IMCL and EMCL of tibialis anterior (ta, mixed type I/II muscle fibers) and soleus muscle (sol, predominantly type I fibers) were quantified using the ^1^H-magnetic resonance spectroscopy (SIEMENS 3.0 T MAGNETOM Verio). A single volume spectroscopy was applied, with the following measurement parameters: TR 3000 ms, TE 20 ms, volume of interest 10 mm × 10 mm × 10 mm, and 48 acquisitions. The water resonance was set to 4.7 ppm, IMCL resonance was set to 1.3 ppm, and EMCL resonance was set to 1.5 ppm. Voxel positon was chosen in the corresponding T1-weighted images such that the smallest voxel did not contain any visible interstitial tissue or fat, while the largest voxel was close to the subcutaneous fat layer [16]. The calculation was performed in the Java-based magnetic resonance user interface (jMRUI) software package.

### 2.5. Statistics

Statistical analyses were performed using SPSS 18.0 software. The data are presented as the mean ± standard deviation (25th and 75th percentiles) for continuous variables. Continuous variables with normal distribution were compared by ANOVA and those without a normal distribution were compared by the Kruskal-Wallis* H* test, if unpaired, and the Mann-Whitney* U* test, if paired. Correlation coefficients were analysed using Pearson's (normally distributed data) or Spearman's correlation (data not normally distributed).

## 3. Results

### 3.1. Characteristics of Study Participants

Baseline characteristics of participants (shown in Table 1) had no differences between the groups in terms of sex distribution, age, BMI, HDL-c, GFR, AST, and blood pressure. Compared with those with NGT, participants with T2DM and IGT had higher blood glucose and HBA1c (*P* value < 0.05). Participants with IGT had higher CHOL and LDL-c compared with those with NGT (*P* value < 0.05), whereas participants with T2DM had higher TG levels compared to those with NGT (*P* value < 0.05).

### 3.2. *M* Value, *β* Cell Function, and Insulin Sensitivity

The insulin sensitivity variables are listed in Table 2. During the insulin clamp, plasma glucose and insulin levels were kept at a steady-state. The *M* value in participants with NGT was significantly higher in those with IGT and T2DM (*P* = 0.001). IGT and T2DM participants were more insulin resistant compared to NGT participants as measured by HOMA-IR (*P* value = 0.001). The IAI and QUICK values of participants with T2DM were significantly lower than those with NGT (*P* value = 0.05).

### 3.3. Body Fat Composition, Hepatocellular Lipid Content, and Skeletal Muscle Fat Content (IMCL and EMCL)

**Figure 1:**
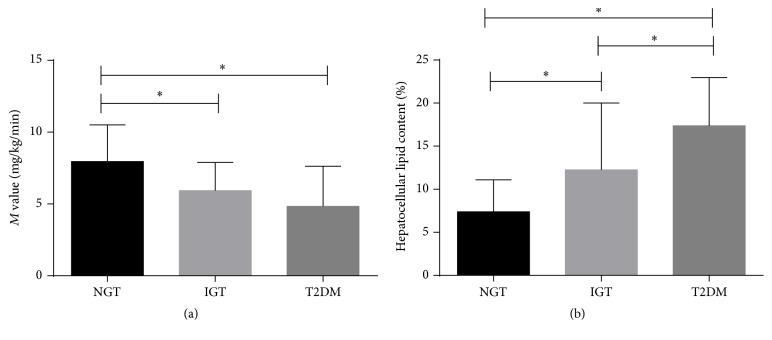
The (a) insulin sensitivity (*M* value) and (b) hepatocellular lipid content in different glucose tolerance status. *∗* refers to the *P* value of comparison between groups which is less than 0.05.

The hepatocellular lipid content of NGT significantly lower than IGT and T2DM participants, and the HCL of IGT significantly lower than T2DM (*P* value < 0.05, results were presented at Table 3, Figure 1(a) (*M* value), and Figure 1(b) (HCL)). The NGT participants with lowest BFR and FM index were compared with IGT and T2DM participants (*P* value < 0.05). Those with T2DM had the highest IMCL (ta) (*P* value = 0.025). The IMCL (sol), EMCL (sol), and EMCL (ta) were similar across the groups.

### 3.4. Correlation Analyses

The *M* value correlated significantly with HCL (*P* value = 0.001), IMCL (sol) (*P* value = 0.002), IMCL (ta) (*P* value = 0.001), and BFR (*P* value = 0.002). Conversely, there was no significant relationship between *M* value and EMCL (sol) or EMCL (ta) (*P* value > 0.05). After adjusting age, sex, and BMI, the correlation of HCL (*P* value = 0.001) and IMCL (ta) (*P* value = 0.028) with *M* value remains significant. See results at Table 4.

### 3.5. Stepwise Linear Regression Analysis

To investigate which of the fat compartments were the strongest determinants of insulin sensitivity, the stepwise linear regression analysis of HCL and skeletal muscle fat content with *M* value was performed after adjusting age, sex, and BMI. HCL (standardized *β* = −0.416; *P* value = 0.001) was independently associated with *M* value, but no statistically significant relationships were observed for IMCL and EMCL.

## 4. Discussion

In T2DM, insulin resistance occurs in different organs, including adipose tissues, liver, and skeletal muscles [17]. Our results state that the HCL and BFR were significantly increased in participants with IGT and T2DM compared with NGT. The IMCL (ta) of T2DM participants were higher than NGT subjects. Therefore, individuals with glucose impairment had greater fat accumulation in the liver and skeletal muscles.

Lipids may accumulate in nonadipose tissues, such as muscles, when the adipose tissues are saturated, thereby causing hepatic and muscle insulin resistance [18]. Meshkani and Adeli [2] showed that hepatic insulin resistance leads to the metabolic syndrome and promotes progression to cardiovascular diseases. Our study showed that HCL, IMCL (sol), IMCL (ta), and BFR correlate inversely with the *M* value, but there was no relationship between *M* value and EMCL. Linear regression analysis demonstrated that HCL is an independent risk factor for dyslipidemia and insulin resistance.

Our findings, consistent with the majority of data, showed that liver fat is the strongest determinant of insulin sensitivity among fat compartments in human [19–22]. Kirchhoff et al. [19] state that insulin sensitivity correlates inversely with hepatocellular lipid content and intramyocellular fat of tibialis anterior muscle, and the study of Kotronen et al. [20] showed that fat accumulation in the liver rather than in skeletal muscle is associated with features of metabolic syndrome, and there was no differences in the IMCL content in those with or without the syndrome. While the data of Krssak et al. [23] showed an inverse correlation between intramyocellular lipid content and *M* value, IMCL is a good indicator of whole-body insulin sensitivity in nondiabetic, nonobese humans.

The discrepancy of studies may be due to methodology differences. Some studies measured the peak area while others measured peak intensity of muscle fat. On measuring liver fat, the hepatic iron metabolism is an important player in the development of systemic insulin resistance [24]. Most of the studies did not measure the hepatic iron load and this may possibly affect the results. The results may also be affected by the exercises or time of measured muscle lipid content [10, 25].

There are several limitations of this study. First, the lack of use of a glucose tracer in the hyperinsulinemic-euglycemic clamp limits the determination of hepatic glucose production rates. Hyperinsulinemic-euglycemic clamp was used to calculate the glucose infusion rates, which is a measure of whole-body glucose disposal and not glucose utilization rates since it does not take into account the hepatic glucose production rates [26]. Second, the sample size was relatively small. Third, we did not include individuals with IFG.

In conclusion, as glucose level increased there was the accumulation of fat deposition. Compared to NGT individuals, IGT and T2DM participants have higher HCL (*P* < 0.05), and the IMCL (ta) of T2DM is significantly higher than NGT (*P* < 0.05). Liver fat deposition happens earlier than skeletal muscle fat deposition. Hepatocellular lipid content plays a predominant role in determination of insulin sensitivity and may be used in evaluating the risk of developing T2DM as a noninvasive marker of insulin sensitivity index.

## Figures and Tables

**Table 1:** Clinical and biochemical characteristics.

	NGT	IGT	T2DM	*P *value
Sex (male/female)	17/12	7/10	6/7	0.484
Age (year)	47.62 ± 8.48	53.24 ± 6.55	50.92 ± 7.32	0.060
BMI (kg/m^2^)	25.14 ± 3.26	25.92 ± 2.96	26.39 ± 2.76	0.441
WHR	0.89 ± 0.07	0.89 ± 0.05	0.94 ± 0.07^†‡^	0.036
SBP (mmHg)	117.45 ± 11.22	123.24 ± 15.77	124.69 ± 14.10	0.181
DBP (mmHg)	74.00 ± 9.17	74.94 ± 8.83	77.85 ± 10.21	0.468
FPG (mmol/L)	5.02 ± 0.45	5.09 ± 0.36	7.48 ± 1.60^†‡^	0.000
PG30 min (mmol/L)	8.87 ± 1.66	10.22 ± 1.65^†^	13.58 ± 2.22^†‡^	0.000
PG2 h (mmol/L)	5.53 ± 1.00	8.97 ± 0.95^†^	15.66 ± 2.89^†‡^	0.000
HBA1c (%)	5.52 ± 0.53	6.06 ± 0.30^†^	7.28 ± 1.23^†‡^	0.000
CHOL (mmol/L)	5.12 ± 0.96	6.04 ± 1.02^†^	5.41 ± 1.07	0.014
TG (mmol/L)	1.19 (0.60–1.75)	1.57 (1.27–1.95)	2.03 (1.44–3.70)^†^	0.002
HDL-c (mmol/L)	1.26 (1.05–1.52)	1.27 (1.07–1.36)	1.05 (0.93–1.26)	0.074
LDL-c (mmol/L)	3.31 ± 0.91	4.37 ± 1.00^†^	3.75 ± 0.96	0.002
eGFR	102.18 ± 24.11	98.08 ± 13.15	98.79 ± 22.00	0.785
(mL/min × 1.73 m^2^)				
ALT (U/L)	19.00 (14.00–25.00)	21.00 (19.00–24.00)	39.50 (27.00–48.00)^†‡^	0.003
AST (U/L)	20.00 (19.00–23.00)	21.00 (19.00–27.00)	28.50 (17.00–33.00)	0.102
GGT (U/L)	23.00 (20.00–35.00)	28.00 (20.00–40.00)	49.00 (34.00–83.00)^†^	0.003

Data are presented as the mean ± SD or median (25th–75th). ^†^NGT versus IGT and T2DM *P* < 0.05, ^‡^IGT versus T2DM *P* < 0.05. BMI: body mass index, WHR: waist-to-hip ratio, SBP: systolic blood pressure, DBP: diastolic blood pressure, FPG: fasting plasma glucose, PG30 min: 30 min plasma glucose, PG2 h: 2 h plasma glucose, HBA1c: hemoglobin A1c, CHOL: total cholesterol, TG: triglyceride, HDL-c: high-density lipoprotein, LDL-c: low-density lipoprotein, ALT: alanine aminotransferase, AST: aspartate aminotransferase, GGT: gamma-glutamyl transpeptidase, and eGFR: estimated glomerular filtration rate.

**Table 2:** Insulin sensitivity and resistance.

	NGT	IGT	T2DM	*P* value
*M* value (mg/kg/min)	7.96 ± 2.54	5.93 ± 1.97^†^	4.83 ± 2.80^†^	0.001
HOMA-IR	1.28 (0.97–1.50)	1.98 (0.93–2.55)	3.14 (2.03–3.32)^†^	0.001
HOMA-*β*	79.70 (61.05–106.25)	122.86 (68.75–148.00)	69.32 (47.80–108.89)	0.233
IAI	0.03 (0.03–0.05)	0.02 (0.02–0.05)	0.01 (0.01–0.02)^†^	0.001
QUICK	0.37 (0.36–0.39)	0.34 (0.33–0.39)	0.32 (0.32–0.34)^†^	0.001

Data are presented as the mean ± SD or median (25th–75th). ^†^NGT versus IGT and T2DM *P* < 0.05. *M* value: Matsuda value calculated by hyperinsulinemic-euglycemic clamp, HOMA-IR: homeostasis assessment insulin resistance, HOMA-*β*: homeostasis assessment-*β*, IAI: insulin activation indices, and QUICK: quantitative insulin sensitivity check index.

**Table 3:** Body fat composition, HCL, and skeletal muscle fat content.

	NGT	IGT	T2DM	*P *value
BFR (%)	25.22 ± 6.19	31.42 ± 6.47^†^	30.63 ± 9.54^†^	0.010
FM index	6.45 ± 2.14	8.24 ± 2.29^†^	8.28 ± 3.15^†^	0.022
LM index	18.77 ± 2.15	17.75 ± 2.20	18.11 ± 1.50	0.253
HCL (%)	7.39 ± 3.71	12.27 ± 7.73^†^	17.37 ± 5.60^†‡^	0.000
IMCL (sol)	7.88 (6.10–10.12)	8.25 (7.04–10.35)	10.70 (9.10–12.25)	0.208
(mmol/kg)				
EMCL (sol)	17.54 (12.32–25.47)	18.62 (10.99–27.41)	22.84 (14.64–44.05)	0.754
(mmol/kg)				
IMCL (ta)	1.74 (1.19–2.55)	2.52 (1.57–3.78)	5.20 (2.89–5.28)^†^	0.025
(mmol/kg)				
EMCL (ta)	4.91 (2.56–9.59)	7.82 (3.93–11.01)	5.46 (2.85–9.48)	0.360
(mmol/kg)				

Data are presented as the mean ± SD or median (25th–75th). ^†^NGT versus IGT and T2DM *P* < 0.05, ^‡^IGT versus T2DM *P* < 0.05. BFR: body fat ratio, FM index: fat mass index, and LM index: lean mass index; hepatocellular lipid content (HCL), intramyocellular lipid (IMCL), and extramyocellular lipid (EMCL) of tibialis anterior (ta) and soleus muscle (sol).

**Table 4:** Association of *M* value with variables.

	Unadjusted		Adjusting sex, age, and BMI	
	*r*	*P* value	*r*	*P* value
BMI	−0.649	0.000		
WHR	−0.377	0.003	−0.273	0.058
SBP	−0.299	0.021	−0.294	0.041
DBP	−0.328	0.011	−0.281	0.050
FPG	−0.341	0.008	−0.211	0.130
PG30 min	−0.410	0.001	−0.381	0.005
PG2 h	−0.498	0.000	−0.473	0.000
HBA1c	−0.357	0.005	−0.242	0.081
CHOL	−0.181	0.171	−0.066	0.640
TG	−0.524	0.000	−0.508	0.000
HDL-c	0.451	0.000	0.501	0.000
LDL-c	−0.250	0.056	−0.177	0.205
GFR	−0.041	0.758	0.113	0.419
ALT	−0.469	0.000	−0.365	0.007
AST	−0.296	0.023	−0.189	0.176
GGT	−0.512	0.000	−0.450	0.001
HOMA-IR	−0.720	0.000	−0.412	0.002
HOMA-*β*	−0.370	0.004	−0.148	0.278
IAI	0.720	0.000	0.067	0.624
QUICK	0.721	0.000	0.211	0.119
BFR	−0.404	0.002	−0.151	0.266
FM index	−0.571	0.000	0.030	0.828
LM index	−0.165	0.213	0.155	0.256
HCL	−0.789	0.000	−0.613	0.000
IMCL (sol)	−0.427	0.002	−0.084	0.577
EMCL (sol)	−0.115	0.430	0.057	0.708
IMCL (ta)	−0.419	0.002	−0.323	0.028
EMCL (ta)	−0.067	0.644	0.074	0.624

Correlation coefficients were analysed using Pearson's (normally distributed data) or Spearman's correlation (data not normally distributed).
